# The discovery, distribution, and diversity of DNA viruses associated with *Drosophila melanogaster* in Europe

**DOI:** 10.1093/ve/veab031

**Published:** 2021-04-01

**Authors:** Megan A Wallace, Kelsey A Coffman, Clément Gilbert, Sanjana Ravindran, Gregory F Albery, Jessica Abbott, Eliza Argyridou, Paola Bellosta, Andrea J Betancourt, Hervé Colinet, Katarina Eric, Amanda Glaser-Schmitt, Sonja Grath, Mihailo Jelic, Maaria Kankare, Iryna Kozeretska, Volker Loeschcke, Catherine Montchamp-Moreau, Lino Ometto, Banu Sebnem Onder, Dorcas J Orengo, John Parsch, Marta Pascual, Aleksandra Patenkovic, Eva Puerma, Michael G Ritchie, Omar Rota-Stabelli, Mads Fristrup Schou, Svitlana V Serga, Marina Stamenkovic-Radak, Marija Tanaskovic, Marija Savic Veselinovic, Jorge Vieira, Cristina P Vieira, Martin Kapun, Thomas Flatt, Josefa González, Fabian Staubach, Darren J Obbard

**Affiliations:** 1 The European Drosophila Population Genomics Consortium (DrosEU); 2 Ashworth Laboratories, Institute of Evolutionary Biology, University of Edinburgh, Charlotte Auerbach Road, Edinburgh EH9 3FL, UK; 3 Department of Entomology, University of Georgia, Athens, GA, USA; 4 Université Paris-Saclay, CNRS, IRD, UMR Évolution, Génomes, Comportement et Écologie, 91198 Gif-sur-Yvette, France; 5 Department of Biology, Georgetown University, Washington, DC, USA; 6 Department of Biology, Section for Evolutionary Ecology, Lund University, Sölvegatan 37, Lund 223 62, Sweden; 7 Division of Evolutionary Biology, Faculty of Biology, Ludwig-Maximilians-Universität München, Planegg, Germany; 8 Department of Cellular, Computational and Integrative Biology, CIBIO University of Trento, Via Sommarive 9, Trento 38123, Italy; 9 Department of Medicine & Endocrinology, NYU Langone Medical Center, 550 First Avenue, New York, NY 10016, USA; 10 Institute of Integrative Biology, University of Liverpool, Liverpool L69 7ZB, UK; 11 UMR CNRS 6553 ECOBIO, Université de Rennes1, Rennes, France; 12 Institute for Biological Research “Sinisa Stankovic”, National Institute of Republic of Serbia, University of Belgrade, Bulevar despota Stefana 142, Belgrade, Serbia; 13 Faculty of Biology, University of Belgrade, Studentski trg 16, Belgrade, Serbia; 14 Department of Biological and Environmental Science, University of Jyväskylä, Finland; 15 National Antarctic Scientific Center of Ukraine, 16 Shevchenko Avenue, Kyiv, 01601, Ukraine; 16 Department of Biology, Genetics, Ecology and Evolution, Aarhus University, Ny Munkegade 116, Aarhus C DK-8000, Denmark; 17 Department of Biology and Biotechnology, University of Pavia, Pavia 27100, Italy; 18 Department of Biology, Faculty of Science, Hacettepe University, Ankara, Turkey; 19 Departament de Genètica, Microbiologia i Estadística and Institut de Recerca de la Biodiversitat (IRBio), Universitat de Barcelona, Barcelona, Spain; 20 Centre for Biological Diversity, St Andrews University, St Andrews HY15 4SS, UK; 21 Research and Innovation Center, Fondazione E. Mach, San Michele all’Adige (TN) 38010, Italy; 22 Centre Agriculture Food Environment, University of Trento, San Michele all’Adige (TN) 38010, Italy; 23 Department of Bioscience, Aarhus University, Aarhus, Denmark; 24 Taras Shevchenko National University of Kyiv, 64 Volodymyrska str, Kyiv 01601, Ukraine; 25 Instituto de Biologia Molecular e Celular (IBMC), University of Porto, Porto, Portugal; 26 Instituto de Investigação e Inovação em Saúde, University of Porto, i3S, Porto, Portugal; 27 Department of Evolutionary Biology and Environmental Studies, University of Zürich, Zürich, Switzerland; 28 Division of Cell & Developmental Biology, Medical University of Vienna, Vienna, Austria; 29 Department of Biology, University of Fribourg, Fribourg CH-1700, Switzerland; 30 Institute of Evolutionary Biology (CSIC-UPF), Barcelona, Spain; 31 Department of Evolution and Ecology, University of Freiburg, Freiburg 79104, Germany

**Keywords:** DNA virus, endogenous viral element, *Drosophila*, nudivirus, galbut virus, filamentous virus, adintovirus, densovirus, bidnavirus

## Abstract

*Drosophila melanogaster* is an important model for antiviral immunity in arthropods, but very few DNA viruses have been described from the family Drosophilidae. This deficiency limits our opportunity to use natural host-pathogen combinations in experimental studies, and may bias our understanding of the *Drosophila* virome. Here, we report fourteen DNA viruses detected in a metagenomic analysis of 6668 pool-sequenced *Drosophila*, sampled from forty-seven European locations between 2014 and 2016. These include three new nudiviruses, a new and divergent entomopoxvirus, a virus related to Leptopilina boulardi filamentous virus, and a virus related to Musca domestica salivary gland hypertrophy virus. We also find an endogenous genomic copy of galbut virus, a double-stranded RNA partitivirus, segregating at very low frequency. Remarkably, we find that Drosophila Vesanto virus, a small DNA virus previously described as a bidnavirus, may be composed of up to twelve segments and thus represent a new lineage of segmented DNA viruses. Two of the DNA viruses, Drosophila Kallithea nudivirus and Drosophila Vesanto virus are relatively common, found in 2 per cent or more of wild flies. The others are rare, with many likely to be represented by a single infected fly. We find that virus prevalence in Europe reflects the prevalence seen in publicly available datasets, with Drosophila Kallithea nudivirus and Drosophila Vesanto virus the only ones commonly detectable in public data from wild-caught flies and large population cages, and the other viruses being rare or absent. These analyses suggest that DNA viruses are at lower prevalence than RNA viruses in *D.melanogaster*, and may be less likely to persist in laboratory cultures. Our findings go some way to redressing an earlier bias toward RNA virus studies in *Drosophila*, and lay the foundation needed to harness the power of *Drosophila* as a model system for the study of DNA viruses.

## 1. Introduction

*Drosophila melanogaster* is one of our foremost models for antiviral immunity in arthropods (Huszart and Imler 2008; Mussabekova, Daeffler, and Imler 2017) and more than 100 *Drosophila*-associated viruses have been reported, including at least thirty that infect *D.melanogaster* (Brun and Plus 1980; Wu et al. 2010; Longdon et al. 2015; Webster et al. 2015, 2016; Medd et al. 2018). These include viruses with positive sense single-stranded RNA genomes (+ssRNA), such as Drosophila C virus, negative sense RNA genomes (−ssRNA), such as Drosophila melanogaster sigmavirus, and double-stranded RNA genomes (dsRNA), such as galbut virus. Many of these viruses are common in laboratory fly cultures and in the wild (Webster et al. 2015). For example, the segmented and vertically transmitted galbut virus is carried by more than 50 per cent of wild-caught adult *D.melanogaster* (Webster et al. 2015; Cross et al. 2020). Overall, more than 20 per cent of wild-caught flies carry multiple RNA viruses, and about one third of laboratory fly lines and almost all *Drosophila* cell cultures are infected by at least one RNA virus (Plus 1978; Brun and Plus 1980; Webster et al. 2015; Shi et al. 2018b). However, in contrast to this wealth of RNA viruses, DNA viruses of *Drosophila* were unknown until relatively recently (Brun and Plus 1980; Huszart and Imler 2008).

The first described DNA virus of a drosophilid was published only ten years ago, after discovery through metagenomic sequencing of wild-caught *Drosophila innubila* (Unckless 2011). This virus is a member the *Nudiviridae*, a lineage of large (120–180 kbp) dsDNA viruses that are best known as pathogens of Lepidoptera and Coleoptera (Harrison et al. 2020), but which have genomic ‘fossil’ evidence across a broad host range (Cheng, Li, and Zhange 2020). Drosophila innubila nudivirus infects several *Drosophila* species in North America, with a prevalence of up to 40 per cent in *D.innubila*, where it can substantially reduce fecundity and lifespan (Unckless 2011; Hill and Unckless 2020). The first reported DNA virus of *D.melanogaster* was a closely related nudivirus reported by Webster *et al.* (2015), and referred to as ‘Kallithea virus’ after a collection location. This virus was also first detected by metagenomic sequencing, but PCR surveys indicate that it is common in wild *D.melanogaster* and *Drosophila simulans* (globally 5% and 0.5%, respectively; Webster et al. 2015). Drosophila Kallithea nudivirus has been isolated for experimental study, and reduces male longevity and female fecundity (Palmer et al. 2018). Consistent with its presumed niche as a natural pathogen of *Drosophila*, this virus encodes a suppressor of *D.melanogaster* NF-kappa B immune signalling (Palmer et al. 2019). Prior to the work described here, the only other reported natural DNA virus infection of a drosophilid was the discovery—again through metagenomic sequencing—of a small number of RNA reads from Invertebrate iridescent virus 31 (IIV31; Armadillidium vulgare iridescent virus) in *Drosophila immigrans* and *Drosophila obscura* (Webster et al. 2016). This virus is known as a generalist pathogen of terrestrial isopods (Piegu et al. 2014), but its presence as RNA (indicative of expression) in these *Drosophila* species suggests that it may have a broader host range.

The apparent dearth of specialist DNA viruses infecting Drosophilidae is notable (Brun and Plus 1980; Huszart and Imler 2008), perhaps because DNA viruses have historically dominated studies of insects such as Lepidoptera (Cory and Myers 2003), and because DNA viruses are well known from other Diptera, including the hytrosaviruses of *Musca* and *Glossina* (Kariithi et al. 2017), densoviruses of mosquitoes (Carlson, Suchman, and Buchatsky 2006), and entomopoxviruses of various Culicomorpha (Lawrence 2011). The lack of native DNA viruses for *D.melanogaster* has practical implications for research, as the majority of experiments have had to utilise viruses that do not naturally infect *Drosophila*, and which have not co-evolved with them (Bronkhorst et al. 2014; West and Silverman 2018; but see Palmer et al. 2019).

It not only remains an open question as to whether the *D.melanogaster* virome is really depauperate in DNA viruses; the prevalence of DNA viruses in *Drosophila*, their phylogenetic diversity, their spatial distribution and temporal dynamics, and their genetic diversity all remain almost unstudied. However, many of these questions can be addressed through large-scale metagenomic sequencing of wild-collected flies. As part of a large *Drosophila* population genomics study using pool-sequencing of wild *D.melanogaster*, we previously reported the genomes of four DNA viruses associated with European *Drosophila* samples collected in 2014 (the DrosEU consortium; Kapun et al. 2020). These included a second *melanogaster*-associated nudivirus (there referred to as ‘Esparto virus’), two densoviruses (‘Viltain virus’ and ‘Linvill road virus’), and two segments of a putative bidnavirus (‘Vesanto virus’). Here we expand our sampling to encompass 167 short-read pool-sequenced samples from a total of 6,668 flies, collected seasonally over 3 years from forty-seven different locations across Europe. We use these population genomic data as a metagenomic source to discover additional DNA viruses, estimate their prevalence in time and space, and quantify levels of genetic diversity.

We complete the genome of a novel and highly divergent entomopoxvirus, identify a further three *Drosophila*-associated nudiviruses, fragments of a novel hytrosavirus most closely related to Musca domestica salivary gland hypertrophy virus, fragments of a filamentous virus distantly related to Leptopilina boulardi filamentous virus, and three polinton-like sequences related to adintoviruses. Our improved assemblies and sampling show that Vesanto virus may be composed of up to twelve segments, and appears to represent a new distinct lineage of multi-segmented ssDNA viruses related to bidnaviruses. We find that two viruses (Drosophila Kallithea nudivirus and Drosophila Vesanto virus) are relatively common in European *D.melanogaster*, but that the majority of DNA viruses appear very rare—most probably appearing once in our sampling.

## 2. Methods

### 2.1 Sample collection and sequencing

A total of 6,668 adult male *Drosophila* were collected across Europe by members of the DrosEU consortium between 19 June 2014 and 22 November 2016, using yeast-baited fruit (Kapun et al. 2020, 2021). There were a total of forty-seven different collection sites spread from Recarei in Portugal (8.4° West) to Alexandrov in Russia (38.7° East), and from Nicosia in Cyprus (36.1° North) to Vesanto in Finland (62.6° North). The majority of sites were represented by more than one collection, with many sites appearing in all 3 years, and several being represented by two collections per year (early and late in the *Drosophila* flying season for that location). After morphological examination to infer species identity, a minimum of thirty-three and maximum of forty male flies (mean 39.8) were combined for each site and preserved in ethanol at −20°C or −80°C for pooled DNA sequencing. Male flies were chosen because, within Europe, male *D.melanogaster* should be morphologically unambiguous. Nevertheless, subsequent analyses identified the occasional presence of the sibling species *D.simulans*, and two collections were contaminated with the distant relatives *Drosophila phalerata* and *Drosophila testacea* (below). Full collection details are provided via figshare repository 10.6084/m9.figshare.14161250, and the detailed collection protocol is provided as supporting material in Kapun *et al* (2020).

To extract DNA, ethanol-stored flies were rehydrated in water and transferred to 1.5-ml well plates for homogenisation using a bead beater (Qiagen Tissue Lyzer II). Protein was digested using Proteinase K, and RNA depleted using RNAse A. The DNA was precipitated using phenol-chloroform-isoamyl alcohol and washed before being air dried and re-suspended in TE. For further details, see the supporting material in Kapun *et al.* (2020). DNA was sequenced in three blocks (2014; most of 2015; 2016 and remainder of 2015) by commercial providers using 151 nt paired end Illumina reads. Block 1 libraries were prepared using NEBNext Ultra DNA Lib Prep-24 and NEBNext Multiplex Oligos, and sequenced on the Illumina NextSeq 500 platform by the Genomics Core Facility of the University Pompeu Fabra (Barcelona, Spain). Block II and III libraries were prepared using the NEBNext Ultra II kit and sequenced on the HiSeq X platform by NGX bio (San Francisco, USA). All raw Illumina read data are publicly available under SRA project accession PRJNA388788.

To improve virus genomes, and following an initial exploration of the Illumina data, we pooled the remaining DNA from four of the collections (samples UA_Yal_14_16, ES_Gim_15_30, UA_Ode_16_47 and UA_Kan_16_57) for long-read sequencing using the Oxford Nanopore Technology ‘Minion’ platform. After concentrating the sample using a SpeedVac (ThermoFisher), we prepared a single library using the Rapid Sequencing Kit (SQK-RAD004) and sequenced it on an R9.4.1 flow cell, subsequently calling bases with Guppy version 3.1.5 (https://community.nanoporetech.com).

### 2.2 Read mapping and identification of contaminating taxa

We trimmed Illumina sequence reads using Trim Galore version 0.4.3 (Krueger 2015) and Cutadapt version 1.14 (Martin 2011). To remove *Drosophila* reads, and to quantify potentially contaminating taxa such as *Wolbachia* and other bacteria, fungi, and trypanosomatids, we mapped each dataset against a combined ‘*Drosophila* microbiome’ reference. This reference comprised the genomes of *D.melanogaster* (Chang and Larracuente 2019), *D.simulans* (Nouhaud 2018), three *Drosophila*-associated *Wolbachia* genomes, sixty-nine other bacteria commonly reported to associate with *Drosophila* (including multiple *Acetobacter*, *Gluconobacter*, *Lactobacillus*, *Pantoea*, *Providencia*, *Pseudomonas*, and *Serratia* genomes), and sixteen microbial eukaryotic genomes (including two *Drosophila*-associated trypanosomatids, a microsporidian, the entomopathogenic fungi *Metarhizium anisopliae*, *Beauveria bassiana*, and *Entomophthora muscae*, and several yeasts associated with rotting fruit; the full list of sequence accessions is provided in figshare repository 10.6084/m9.figshare.14161250). All mapping was performed using Bowtie 2 version 2.3.4 or version 2.4.1 (Langmead and Salzberg 2012) and we recorded only the best mapping position for each read, based on alignment match score (the Bowtie 2 default). To provide approximate quantification we used raw mapped read counts, normalised by target length and fly read counts where appropriate.

During manual examination of *de novo* assemblies (below) we identified a number of short contigs from other taxa, including additional species of *Drosophila*, *Drosophila* commensals such as mites and nematodes, and potential sequencing contaminants such as humans and model organisms. To quantify this potential contamination, we re-mapped all trimmed read pairs to a reference panel of short diagnostic sequences. This panel comprised a region of *Cytochrome Oxidase I* (COI) from each of the following: twenty species of *Drosophila* (European *Drosophila* morphologically similar to *D.melanogaster*; and *Drosophila* species identified in *de novo* assemblies); 667 species of nematode (including lineages most likely to be associated with *Drosophila*, and a contig identified by *de novo* assembly); 106 parasitic wasps (including many lineages commonly associated with *Drosophila*); two species of mite (identified in *de novo* assemblies); six model vertebrates; and complete plastid genomes from eight crop species. Because cross-mapping between *D.melanogaster* and *D.simulans* is possible at many loci, we also included a highly divergent but low-diversity 2.3 kbp region of the single-copy gene *Argonaute-2* to estimate levels of *D.simulans* contamination. Where reads indicated the presence of other *Drosophila* species, this was further confirmed by additional mapping to *Adh*, *Amyrel*, *Gpdh*, and *6-PGD*. A full list of the reference sequences is provided in via figshare repository 10.6084/m9.figshare.14161250.

### 2.3 Virus genome assembly and annotation

To identify samples containing potentially novel viruses, we retained read pairs that were not concordantly mapped to the combined ‘*Drosophila* microbiome’ reference (above) and used these for *de novo* assembly using SPAdes version 3.14.0 with the default spread of k-mer lengths (Nurk et al. 2013), after *in silico* normalisation of read depth to a target coverage of 200 and a minimum coverage of three using bbnorm (https://sourceforge.net/projects/bbmap/). We performed normalisation and assembly separately for each of the 167 samples. We then used the resulting scaffolds to search a database formed by combining the NCBI ‘refseq protein’ database with the viruses from NCBI ‘nr’ database. The search was performed using Diamond blastx (version 0.9.31; Buchfink, Xie, and Huson 2014) with an e-value threshold of 1 × 10^−30^, permitting frameshifts, and retaining hits within 5 per cent of the top hit.

The resulting sequences were examined to exclude all phage, retroelements, giant viruses (i.e. mimiviruses and relatives), and likely contaminants such as perfect matches to well-characterised plant, human, pet, and vertebrate livestock viruses (e.g. Ebola virus, Hepatitis B virus, Bovine viral diarrhoea virus, Murine leukaemia virus). We also excluded virus fragments that co-occurred across samples with species other than *Drosophila*, such as mites and fungi, as likely to be viruses of those taxa. Our remaining candidate virus list included known and potentially novel DNA viruses, and one previously reported *Drosophila* RNA virus. For each of these viruses we selected at least one representative population sample, based on high coverage, for targeted genome re-assembly.

For targeted re-assembly of each virus we re-mapped all non-normalised reads to the putative virus scaffolds from the first assembly and retained all read pairs for which at least one partner had mapped. Using these virus-enriched read sets we then performed a second *de novo* SPAdes assembly for each target sample (as above), but to aid scaffolding and repeat resolution we additionally included the long reads (Antipov et al. 2015) that had been generated separately from UA_Yal_14_16, ES_Gim_15_30, UA_Ode_16_47 and UA_Kan_16_57. We examined the resulting assembly graphs using Bandage version 0.8.1 (Wick et al. 2015), and based on inspection of coverage and homology with related viruses we manually resolved short repeat regions, bubbles associated with polymorphism, and long terminal repeat regions. For viruses represented by very few low-coverage fragments, we concentrated assembly and manual curation on genes and gene fragments that would be informative for phylogenetic analysis.

For Drosophila Vesanto virus, a bidna-like virus with two previously reported segments (Kapun et al. 2020), a preliminary manual examination of the assembly graph identified a potential third segment. We therefore took two approaches to explore the possibility that this virus is composed of more than two segments. First, to identify completely new segments, we mapped reads from samples with or without segments S01 and S02 to all high-coverage scaffolds from one sample that contained those segments. This allowed us to identify possible further segments based on their pattern of co-occurrence across samples (e.g. Batson et al. 2020; Obbard et al. 2020). Second, to identify substantially divergent (but homologous) alternative segments we used a blastp similarity search using predicted Vesanto virus proteins and predicted proteins from *de novo* scaffolds (e-value 10^−20^). Again, we examined targeted assembly graphs using Bandage (Wick et al. 2015), and resolved inverted terminal repeats and apparent mis-assemblies manually.

To annotate viral genomes with putative coding DNA sequences we used getORF from the EMBOSS package (Rice, Longden, and Bleasby 2000) to identify all open reading frames of 150 codons or more that started with ATG, and translated these to provide putative protein sequences. We retained those with substantial similarity to known proteins from other viruses, along with those that did not overlap longer open reading frames.

### 2.4 Presence of DNA viruses in publicly available *Drosophila* datasets

To detect all known and novel *Drosophila* DNA viruses present in publicly available DNA *Drosophila* datasets, we chose twenty-eight ‘projects’ from the NCBI Sequence Read Archive and mapped these to virus genomes using Bowtie 2 (Langmead and Salzberg 2012). Among these were several projects associated with the *D.melanogaster* Genome Nexus (Lack et al. 2015; Lange et al. 2016; Sprengelmeyer et al. 2020), the Drosophila Real-Time Evolution Consortium (Dros-RTEC; Machado et al. 2019; Kapun et al. 2021), pooled genome-wide association studies (GWAS; e.g. Endler et al. 2018), evolve-and-resequence studies (Jalvingh et al. 2014; Schou et al. 2017; Kelly and Hughes 2019), studies of local adaptation (e.g. Campo et al. 2013; Kang et al. 2019), and introgression (Kao et al. 2015). In total this represented 3,003 Illumina sequencing ‘run’ datasets. The ‘project’ and ‘run’ identifiers are listed in figshare repository 10.6084/m9.figshare.14161250 file S7. For each run, we mapped up to 10 million reads to *Drosophila* DNA viruses (forward reads only for paired-end datasets), and recorded the best-mapping location for each read, as above. Short reads and low complexity regions allow some cross-mapping among the larger viruses, and between viruses and the fly genome. We therefore chose an arbitrary detection threshold of 250 mapped reads to define the presence of each of the larger viruses (expected genome size >100 kbp) and a threshold of twenty-five reads for the smaller viruses (genome size <100 kbp). Consequently, our estimates may be conservative tests of virus presence, and the true prevalence may be slightly higher. We additionally selected a subset of the public datasets for *de novo* assemblies of Drosophila Vesanto virus (datasets ERR705977, ERR173251, ERR2352541, and SRR3939080), an adintovirus (SRR3939056), and galbut virus (SRR5762793 and SRR1663569), using the same assembly approach as outlined for DrosEU data above.

### 2.5 Phylogenetic inference

To infer the phylogenetic relationships among DNA viruses of *Drosophila* and representative viruses of other species, we selected a small number of highly conserved virus protein-coding loci that have previously been used for phylogenetic inference. For densoviruses we used the viral replication initiator protein, NS1 (Pénzes et al. 2020), for adintoviruses and bidnaviruses we used DNA Polymerase B (Krupovic and Koonin 2014; Starrett et al. 2020), for Poxviruses we used rap-94, and the large subunits of Poly-A polymerase and the mRNA capping enzyme (Thézé et al. 2013), and for nudiviruses, filamentous viruses and hytrosaviruses we used P74, Pif-1, Pif-2, Pif-3, Pif-5 (ODV-e56), and the DNA polymerase B (e.g., Kawato et al. 2018). In each case we used a blastp search to identify a representative set of similar proteins in the NCBI ‘nr’ database, and among proteins translated from publically available transcriptome shotgun assemblies deposited in GenBank. For the nudiviruses, filamentous viruses and hytrosaviruses we combined these with proteins collated by Kawato *et al* (2018). We aligned protein sequences for each locus using t-coffee mode ‘accurate’, which combines structural and profile information from related sequences (Notredame, Higgins, and Heringa 2000), and manually trimmed poorly aligned regions from each end of each alignment. We did not filter the remaining alignment positions for coverage or alignment ‘quality’, as this tends to bias toward the guide tree and to give false confidence (Tan et al. 2015). We then inferred trees from concatenated loci (where multiple loci were available) using IQtree2 with default parameters (Minh et al. 2020), including automatic model selection and 1,000 ultrafast bootstraps.

### 2.6 Age of an endogenous viral element

To infer the age of an endogenous copy (endogenous viral element [EVE]) of galbut virus (a dsRNA partitivirus; Cross et al. 2020), we used a strict-clock Bayesian phylogenetic analysis of virus sequences, as implemented in BEAST 1.10.2 (Suchard et al. 2018). To make this inference our assumption is that any evolution of the EVE after insertion is negligible relative to RNA virus evolutionary rates. We assembled complete 1.6 kb segment sequences from publicly available RNA sequencing datasets (Lin et al. 2016; Garlapow et al. 2017; Yablonovitch et al. 2017; Bost et al. 2018; Shi et al. 2018b; Everett et al. 2020), and filtered these to retain unique sequences and exclude possible recombinants identified with GARD (Kosakovsky Pond et al. 2006). The few recombinants were all found in multiply infected pools, suggesting they may have been chimeric assemblies. For sequences from Shi et al. (2018b) we constrained tip dates according to the extraction date, and for other studies we constrained tip dates to the 3-year interval prior to project registration. We aligned these sequences with the EVE sequence, and during phylogenetic analysis we constrained most recent date for the EVE to be its extraction date, but left the earliest date effectively unconstrained. Because the range of virus tip dates covered <10 years we imposed time information through a strongly informative log-normal prior on the strict clock rate, chosen to reflect the spread of credible evolutionary rates for RNA viruses (e.g. Peck and Lauring 2018). Specifically, we applied a data-scale mean evolutionary rate of 4 × 10^−4^ events/site/year with standard deviation 2.5 × 10^−4^, placing 95 per cent of the prior density between 1 × 10^−3^ and 1.3 × 10^−4^. As our sampling strategy was incompatible with either a coalescent or birth-death tree process, we used a Bayesian Skyline coalescent model to allow flexibility in the coalescence rate, and thereby minimise the impact of the tree prior on the date (although alternative models gave qualitatively similar outcomes). We used the SDR06 substitution model (Shapiro, Rambaut, and Drummond 2006) and otherwise default priors, running the Monte-Carlo Markov Chain for 100 million steps and retaining every 10,000th state. The effective sample size was >1,400 for every parameter. BEAST input xml is provided via figshare repository 10.6084/m9.figshare.14161250.

### 2.7 Virus quantification, and the geographic and temporal distribution of viruses

To quantify the (relative) amount of each virus in each pooled sample, we mapped read pairs that had not been mapped concordantly to the *Drosophila* microbiome reference (above) to the virus genomes. This approach means that low complexity reads map initially to the fly and microbiota, and are thus less likely to be counted or mis-mapped among viruses. This slightly reduces the detection sensitivity (and counts) but also increases the specificity. We mapped using Bowtie 2 (Langmead and Salzberg 2012), recording the best mapping location as above. We used either read count (per million reads) divided by target length (per kilobase) to quantify the viruses, or this value normalised by the equivalent number for *Drosophila* (combined *D.melanogaster* and *D.simulans* reads) to provide an estimate of virus genomes per fly genome in each pool. To quantify Vesanto virus genomes we excluded terminal inverted repeats from the reference, as these may be prone to cross-mapping among segments.

To provide a simple estimate of prevalence, we assumed that pools represented independent samples from a uniform global population, and assumed that a pool of *n* flies constituted *n* Bernoulli trials in which the presence of virus reads indicated at least one infected fly (e.g. Speybroeck et al. 2012). Based on this model, we inferred a maximum-likelihood estimate of global prevalence for each virus, with 2 log-likelihood intervals. Because some cross-mapping between viruses is possible, and because barcode switching can cause reads to be misassigned among pools, we chose to use a virus detection threshold of 1 per cent of the fly genome copy number to define ‘presence’. This threshold was chosen on the basis that male flies artificially infected with Drosophila Kallithea nudivirus have a virus genome copy number five-fold higher than that of the fly three days post infection (Palmer et al. 2018), or around 1 per cent of the fly genome copy number for a single infected fly in a pool of 40. Thus, although our approach may underestimate virus prevalence if titre is low, it provides some robustness to barcode switching while also giving reasonable power to detect a single infected fly.

In reality, pools are not independent of each other in time or space, or other potential predictors of viral infection. Therefore, for the three most prevalent viruses (Drosophila Kallithea nudivirus, Drosophila Linvill Road densovirus, and Drosophila Vesanto virus) we analysed predictors of the presence and absence of each virus within population pools using a binomial generalised linear mixed model approach. We fitted linear mixed models in a spatial framework using R-INLA (Blangiardo et al. 2013), taking a Deviance Information Criterion (DIC) of two or larger as support for a spatial or spatiotemporal component in the model. In addition to any spatial random effects, we included one other random-effect and four fixed-effect predictors. The fixed effects were: the level of *D.simulans* contamination (measured as the percentage *D.simulans* Ago2 reads); the amount of *Wolbachia* (measured as reads mapping to *Wolbachia* as relative to the number mapped to fly genomes); the sampling season (early or late); and the year (unordered categorical 2014–16). We included sampling location as a random effect, to account for any additional non-independence between collections made at the same sites or by the same collector. The inclusion of a spatially distributed random effect was supported for Drosophila Kallithea nudivirus and Drosophila Linvill Road densovirus, but this did not vary significantly with year. Map figures were plotted and model outputs summarised with the R package ggregplot (https://github.com/gfalbery/ggregplot), and all code to perform these analyses is provided via figshare repository 10.6084/m9.figshare.14161250.

### 2.8 Virus genetic diversity

Reads that had initially been mapped to Drosophila Kallithea nudivirus, Drosophila Linvill Road densovirus and Drosophila Vesanto virus (above) were remapped to reference virus genomes using BWA-MEM with local alignment (Li 2013). For the segmented Drosophila Vesanto virus, we included multiple divergent haplotypes in the reference but excluded terminal inverted repeats, as reads derived from these regions will not map uniquely. After identifying the most common haplotype for each Drosophila Vesanto virus segment in each of the samples, we remapped reads to a single reference haplotype per sample. For all viruses, we then excluded secondary alignments, alignments with a Phred-scaled mapping quality (MAPQ) <30, and optical and PCR duplicates using picard v.2.22.8 ‘MarkDuplicates’ (http://broadinstitute.github.io/picard/). Finally, we excluded samples that had a read-depth of <25 across 95 per cent of the mapped genome.

In addition to calculating per-sample diversity, to calculate total population genetic diversity we created single global pool, representative of diversity across the whole population, by merging sample bam files for each virus or segment haplotype. To reduce computational demands, each was down-sampled to an even coverage across the genome (no greater read depth at a site than the original median) and no sample contributed more than 500-fold coverage. To produce the final dataset for analyses, bam files for the global pool and each of the population pools were re-aligned around indels using GATK v3.8 (Van der Auwera et al. 2013). We created mPileup files using SAMtools (Li et al. 2009) to summarise each of these datasets using (minimum base quality = 40 and minimum MAPQ = 30), down-sampling population samples to a maximum read depth of 500. We masked regions surrounding indels using ‘popoolation’ (Kofler et al. 2011b), and generated allelic counts for variant positions in each using ‘popoolation2’ (Kofler, Pandey, and Schlötterer 2011a), limiting our search to single nucleotide polymorphisms (SNPs) with a minor allele frequency (MAF) of at least 1 per cent.

To calculate average pairwise nucleotide diversity at synonymous (π_S_) and non-synonymous (π_A_) sites we identified synonymous and non-synonymous SNPs using popoolation (Kofler et al. 2011b), excluding SNPs with a MAF of <1 per cent. In general, estimates of genetic diversity from pooled samples, such as those made by popoolation and popoolation2, attempt to account for variation caused by finite sample sizes of individuals each contributing to the pool of nucleic acid. However, such approaches cannot be applied to viruses from pooled samples, as it is not possible to infer the number of infected flies in the pool or even to equate an infected fly with an individual (flies may be multiply infected). For this reason, we calculated π_A_ and π_S_ based on raw allele counts derived from read frequencies (code is provided via figshare repository 10.6084/m9.figshare.14161250). We did this separately for each gene in the merged global pool, and also for the whole genome in each infected population pool.

### 2.9 Structural variation and indels in Drosophila Kallithea nudivirus

Large DNA viruses such as Drosophila Kallithea nudivirus can harbour transposable element (TE) insertions and structural rearrangements (Loiseau et al. 2020), and often contain abundant length variation in short repeats (Zhao et al. 2012). To identify large-scale rearrangements, we identified all read pairs for which at least one read mapped to Drosophila Kallithea nudivirus, and used SPAdes (Bankevich et al. 2012) to perform *de novo* assemblies separately for each dataset using both *in silico* normalised and un-normalised reads. We then selected those scaffolds approaching the expected length of the genome (>151 Kbp), and examined the assembly graphs manually using bandage (Wick et al. 2015), retaining those in which a single circular scaffold could be seen, with a preference for un-normalised datasets. These were then linearised starting at the DNA Polymerase B coding sequence, and aligned using muscle (Edgar 2004). This approach will miss structural variants at low frequency within each population, but could identify any major rearrangements that are fixed differently across populations.

To detect polymorphic TE insertions that were absent from the reference genome, we identified sixteen population samples that had more than 300-fold read coverage of Drosophila Kallithea nudivirus and extracted all reads that mapped to the virus. We aligned these to 135 *D.melanogaster* TEs curated in the November 2016 version of Repbase (Bao, Kojima, and Kohany 2015) using blastn (-task megablast). All reads for which one portion aligned to the virus (Genome reference KX130344.1) and another portion aligned to a *D.melanogaster* TE were identified as chimeric using the R script provided by Peccoud *et al.* (2018), and those for which the read-pair spanned TE ends were considered evidence of a TE insertion.

Finally, to catalogue short indel polymorphisms in coding and intergenic regions, we used popoolation2 (Kofler, Pandey, and Schlötterer 2011a) to identify the genomic positions (relative to the reference genome) in each of the infected samples for which a gap was supported by at least five reads. We used a chi-square test for independence to test if there was an association between the coding status of a position and the probability that an indel was supported at that position in at least one population sample.

## 3 Results

Over six and a half thousand flies were collected from forty-seven locations across Europe across three years as part of the DrosEU project (Kapun et al. 2020, 2021). Their DNA was sequenced in population pools of around forty flies, resulting in a total of 8.4 billion trimmed read pairs, with between 27.3 and 78 million pairs per sample. Using these reads we find evidence for fourteen distinct DNA viruses associated with *D.melanogaster* in Europe, of which nine have not been previously reported. We find two of the viruses to occur at a relatively high prevalence of 2–3 per cent, but most are extremely rare.

### 3.1 Host species composition

On average, 93 per cent of reads (range 70–98%) could be mapped to *Drosophila* or likely components of the *Drosophila* microbial community. *Wolbachia* made up an average of 0.5 per cent of mapped non-fly reads (range 0.0–2.9%); other mapped bacterial reads together were 0.6 per cent (0.0–3.2%), and microbial eukaryotes were 0.3 per cent (0.0–3.7%). The eukaryotic microbiota included the fungal pathogen *E.muscae* (e.g. Elya et al. 2018), with reads present in forty-two of 167 samples (up to 1.38 reads per kilobase per million reads, RPKM), a novel trypanosomatid distantly related to *Herpetomonas muscarum* (e.g. Sloan et al. 2019) with reads present in eighty samples (up to 0.87 RPKM). We also identified the microsporidian *Tubulinosema ratisbonensis* (e.g. Niehus et al. 2012) in one sample (0.54 RPKM). We excluded two virus-like DNA Polymerase B fragments from the analyses because they consistently co-occurred with a fungus very closely related to *Candida* (*Clavispora*) *lusitaniae* (correlation coefficient on >0.94, *p *<* *10^−10^; figshare repository 10.6084/m9.figshare.14161250 File S4). For a detailed assessment of the microbial community in the 2014 collections, see Kapun *et al* (2020) and Wang *et al* (2020). Raw and normalised read counts are presented in figshare repository 10.6084/m9.figshare.14161250 File S3, and raw data are available from the Sequence Read Archive under project accession PRJNA388788.

The remaining 2–30 per cent of reads could include metazoan species associated with *Drosophila*, such as nematodes, mites, or parasitoid wasps. By mapping all reads to small reference panel of COI sequences, we identified thirteen samples with small read numbers mapping to potentially parasitic nematodes, including an unidentified species of *Steinernema*, two samples with reads mapping to *Heterorhabditis bacteriophora* and three with reads mapping to *Heterorhabditis marelatus*. *De novo* assembly also identified an 8.4 kbp nematode scaffold with 85 per cent nucleotide identity to the mitochondrion of *Panagrellus redivivus*, a free-living rhabditid associated with decomposing plant material. Reads from this nematode were detectable in seventy-three of the 167 samples, rarely at a high level (up to 0.8 RPKM). Only one sample contained reads that mapped to mite COI, sample UK_Dai_16_23, which mapped at high levels (5.8 and 2.2 RPKM) to two unidentified species of Parasitidae (Mesostigmata, Acari). We excluded two cyclovirus-like fragments from the analyses below because they occurred only in the sample contaminated with the two mites, suggesting that they may be associated with the mites or integrated into their genomes (figshare repository 10.6084/m9.figshare.14161250 S3 and S4).

To detect the presence of drosophilid hosts other than *D.melanogaster*, we mapped all reads to a curated panel of short diagnostic sequences from *COI* and *Argonaute-2*, the latter chosen for its ability to reliably distinguish between the close relatives *D.melanogaster* and *D.simulans*. As expected from previous analyses of these data (e.g. Kapun et al. 2020), thirty of the 167 samples contained *D.simulans* at a threshold of >1 per cent of *Ago2* reads. Mapping to *COI* sequences from different species, we identified only three further *Drosophila* species present in any sample at a high level. These included two small yellowish European species; *D.testacea*, which accounted for 2.4 per cent of COI in UA_Cho_15_26 (263 reads), and *D.phalerata*, which accounted for 12.2 per cent of COI in AT_Mau_15_50 (566 reads). The presence of both species was confirmed by additional mapping to *Adh*, *Amyrel*, *Gpdh*, and *6-PGD* (figshare repository 10.6084/m9.figshare.14161250 S3), and their mitochondrial genomes were recovered as 9 and 16 kbp *de novo* scaffolds, respectively. More surprisingly, some of the collections made in 2015 contained reads derived from *D.serrata*, a well-studied species closely related to *D.melanogaster* and endemic to tropical Australia (Reddiex, Allen, and Chenoweth 2018). Samples TR_Yes_15_7 and FR_Got_15_48 had particularly high levels of *D.serrata COI*, with 94 per cent (23,911 reads) and 7 per cent (839 reads) of *COI* respectively, but reads were also detectable in another six pools. The presence of *D.serrata* sequences was confirmed by mapping to *Adh*, *Amyrel*, *Gpdh*, and *6-PGD* (figshare repository 10.6084/m9.figshare.14161250 S3). However, examination of splice junctions showed that *D.serrata* reads derived from cDNA rather than genomic DNA, and must therefore result from cross-contamination during sequencing or from barcode switching. Below, we note where conclusions may be affected by the presence of species of other than *D.melanogaster*.

Finally, among *de novo* assembled contigs, we also found evidence for several crop-plant chloroplasts and vertebrate mitochondria that are likely to represent sequencing or barcode-switching contaminants. The amounts were generally very low (median 0.01 RPKM), but a few samples stood out as containing potentially high levels of these contaminants. Most notably sample TR_Yes_15_7, in which only 76 per cent of reads mapped to fly or expected microbiota, had 8.1 RPKM of human mtDNA, 5.1 RPKM of *Cucumis melo* chloroplast DNA, and 3.5 RPKM of *Oryza sativa* chloroplast DNA. We do not believe contamination of this sample has any impact on our findings.

### 3.2 Previously reported DNA virus genomes

**Figure 1.**
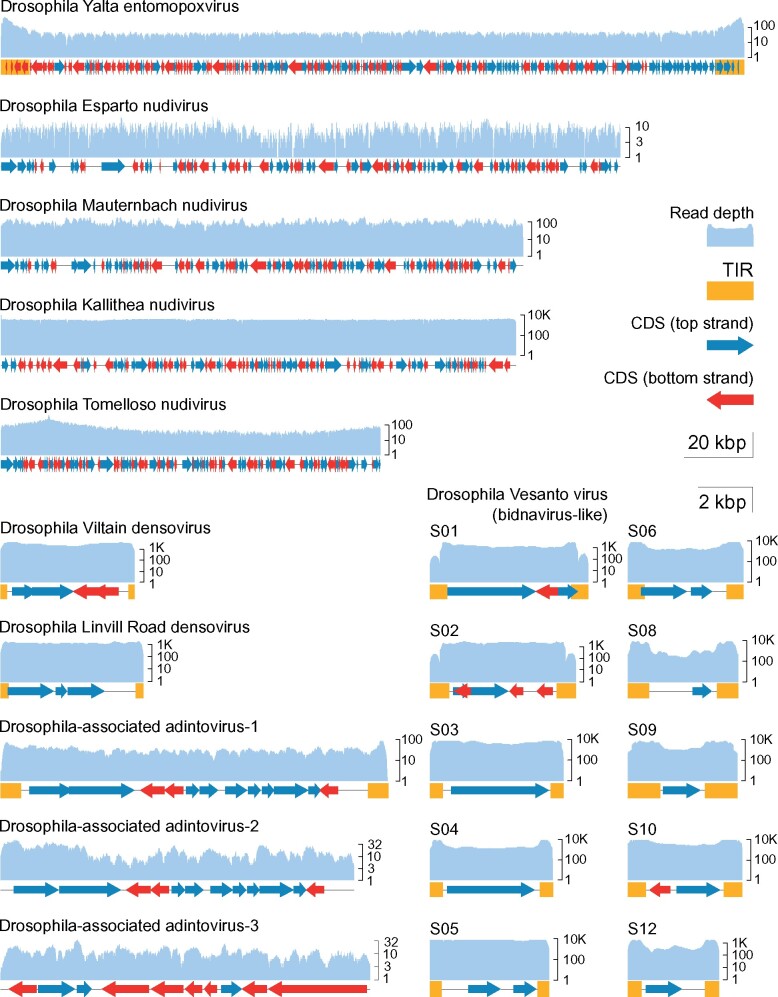
Genome structures and read depth. The plots show annotated coding DNA sequences (CDS, red and blue arrows), and terminal Inverted repeat (yellow boxes) for each of the near-complete virus genomes discussed. The read depth (pale blue) is plotted above the genome on a log scale for the population with the highest coverage in the DrosEU dataset. The five largest viruses (top) are plotted according to the 20 kbp scale bar, and the other viruses (bottom) are plotted according to the 2 kbp scale bar. The nudiviruses are circular, and have been arbitrarily linearised for plotting. Drosophila Esparto nudivirus was completed using public dataset (SRR3939042). Note that Drosophila Vesanto virus segments S07 and S11 were absent from the illustrated sample (lower right).

**Figure 2.**
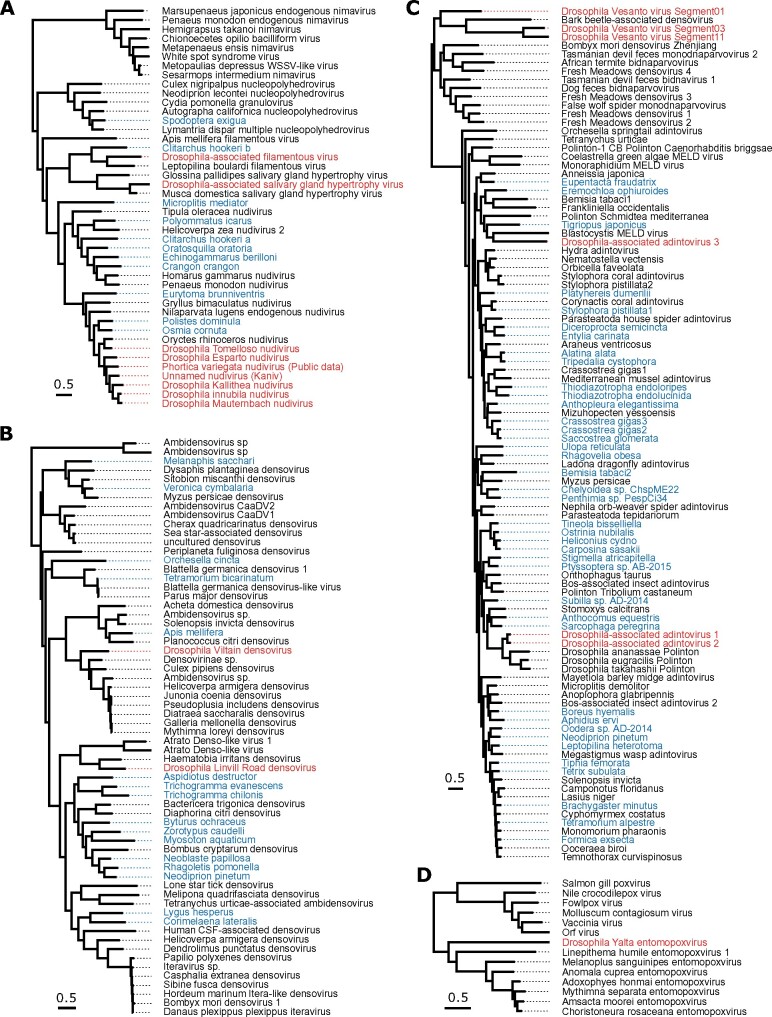
Phylogenetic relationships. (A) Nudiviruses, hytrosaviruses, filamentous viruses, nucleopolyhedrosis viruses and nimaviruses, inferred from six concatenated protein coding genes. Note that these lineages are extremely divergent, and the alignment is not reliable at deeper levels of divergence. (B) Densoviruses, inferred from NS1. (C) Bidnaviruses (sometimes labelled ‘densovirus’) and adintoviruses (including representative polintons), inferred from DNA Polymerase B. (D) Pox and entomopoxviruses, inferred from three concatenated protein coding genes. All phylogenies were inferred from protein sequences by maximum likelihood, and scale bars represent 0.5 amino-acid substitutions per site. In each case, trees are mid-point rooted, viruses reported from *Drosophila* are shown in red, and sequences identified from virus transcripts in publicly available transcriptome assemblies are shown in blue, labelled by host species. The nudivirus from *Phortica variegata* was derived from PRJNA196337 (Vicoso and Bachtrog 2013). Alignments and tree files with bootstrap support are available in Supplementary Material.

Six different DNA viruses were previously detected among DrosEU samples from 2014 and reported by Kapun *et al.* (2020). These included one known virus (Drosophila Kallithea nudivirus; Webster et al. 2015) and five new viruses, of which four were assembled by Kapun *et al.* (2020). Drosophila Kallithea nudivirus is a relatively common virus of *D.melanogaster* (Webster et al. 2015) that has a circular dsDNA genome of *ca.* 153 kbp encoding ∼95 proteins (Fig. 1), and is closely related to Drosophila innubila nudivirus (Fig. 2A). Drosophila Esparto nudivirus is a second nudivirus associated with *D.melanogaster* that was present at levels too low to permit assembly by Kapun *et al.* (2020), but was instead assembled in that paper from a *D.melanogaster* sample collected in Esparto, California USA (SRA dataset SRR3939042; Machado et al. 2019). It has a circular dsDNA genome of *ca.* 183 kbp that encodes ∼90 proteins, and it is closely related to Drosophila innubila nudivirus and Drosophila Kallithea nudivirus (Figs 1 and 2A). Drosophila Viltain densovirus and Drosophila Linvill Road densovirus are both small viruses related to parvoviruses, with ssDNA genomes of ∼5 kb. Drosophila Viltain densovirus is most closely related to Culex pipiens ambidensovirus (Jousset, Baquerizo, and Bergoin 2000), and the genome appears to encode at least four proteins—two in each orientation (Figs 1 and 2B). As expected, the ends of the genome are formed of short-inverted terminal repeats (Fig. 1). Drosophila Linvill Road densovirus is most closely related to the unclassified Haemotobia irritans densovirus (Ribeiro et al. 2019) and appears to encode at least three proteins, all in the same orientation (Figs 1 and 2B). As with Drosophila Esparto nudivirus, Kapun *et al.* (2020) were unable to assemble the Drosophila Linvill Road densovirus genome from the DrosEU 2014 data and instead based their assembly on a collection of *D.simulans* from Linvilla, Pennsylvania, USA (SRR2396966; Machado et al. 2019). Here we identified a DrosEU 2016 collection (ES_Ben_16_32; Benalua, Spain) with sufficiently high titre to permit an improved genome assembly (submitted to GenBank under accession MT490308). This is 99 per cent identical to the previous Drosophila Linvill Road densovirus assembly, but by examination of the assembly graph we were able to complete more of the inverted terminal repeats and extend the genome length to 5.4 kb (Fig. 1). Table 1 provides a summary of all DNA viruses detectable in DrosEU data.

**Table 1.** DNA Viruses of *Drosophila* present in the DrosEU dataset.

Virus name	Relationship	Genome status	Assembly (bp)	First published	Genbank accessions	DrosEU code	Genome collection location	Source data
Drosophila Kallithea nudivirus	Nudiviruses	Complete	152,388	Webster et al. (2015)	KX130344	UA_Kha_14_46	Kharkiv, Ukraine	SRR5647730
Drosophila Esparto nudivirus	Nudiviruses	Complete	183,261	Kapun et al. (2020)	KY608910	(Dros-RTEC)	Esparto, California	SRR3939042
Drosophila Viltain densovirus	Densoviruses	Complete	5,025	Kapun et al. (2020)	KX648535	FR_Vil_14_07	Viltain, France	SRR5647729
Drosophila Linvill Road densovirus	Densoviruses	Complete	5,360	Kapun et al. (2020) Expanded here	MT490308	ES_Ben_16_32	Benalua, Spain	SRR8494475
Drosophila Vesanto virus	Bidna-like viruses	Up to twelve Segments	Up to 52,097	Kapun et al. (2020) expanded here	MT496850– MT496878	UA_Kan_16_57 UA_Dro_16_56 FR_Got_15_48	Kaniv, Ukraine; Drogobych, Ukraine; and Gotheron, France	SRR8494448 SRR8494441 SRR8439123
Drosophila Tomelloso nudivirus	Nudiviruses	Complete	112,307	This article	KY457233	ES_Tom_15_28	Tomelloso, Spain	SRR8439136
Drosophila Mauternbach nudivirus	Nudiviruses	Complete	154,465	This article	MG969167	AT_Mau_15_50	Mauternbach, Austria	SRR8439127
Drosophila Yalta entomopoxvirus	Entomopoxviruses	Complete	219,929	This article	MT364305	UA_Yal_14_16	Yalta, Ukraine	SRR5647764
Drosophila-associated nudivirus (Kaniv)	Nudiviruses	Fragmentary	4,503	This article	MT496841– MT496846	UA_Kan_16_57	Kaniv, Ukraine	SRR8494448
Drosophila-associated filamentous virus	Filamentous viruses	Fragmentary	86,478	This article	MT496832– MT496840	ES_Gim_15_30	Gimenells, Spain	SRR8439138
Drosophila-associated hytrosavirus	Hytrosaviruses	Fragmentary	16,606	This article	MT469997– MT470014	UA_Ode_16_47	Odesa, Ukraine	SRR8494427
Drosophila-associated adintovirus 1	Adintoviruses	Complete	14,567	This article	MT496847	UA_Cho_15_26	Kopachi (Chornobyl Exclusion Zone), Ukraine	SRR8439134
Drosophila-associated adintovirus 2	Adintoviruses	Near-complete	13,277	This article	MT496848	AT_Mau_15_50	Mauternbach, Austria	SRR8439127
Drosophila-associated adintovirus 3	Adintoviruses	Near-complete	13,883	This article	MT496849	DK_Kar_16_4	Karensminde, Denmark	SRR8494437

### 3.3 Drosophila Vesanto virus may be a multi-segmented bidna-like virus

Kapun *et al.* (2020) also reported two segments of a putative ssDNA bidnavirus, there called ‘Vesanto virus’ for its collection site in 2014 (submitted to GenBank in 2016 as KX648533 and KX648534). This was presumed to be a complete genome based on homology with Bombyx mori bidensovirus (Li et al. 2019). Here, we have been able to make use of expanded sampling and a small number of long-read sequences to extend these segments and to identify multiple co-occurring segments.

**Figure 3.**
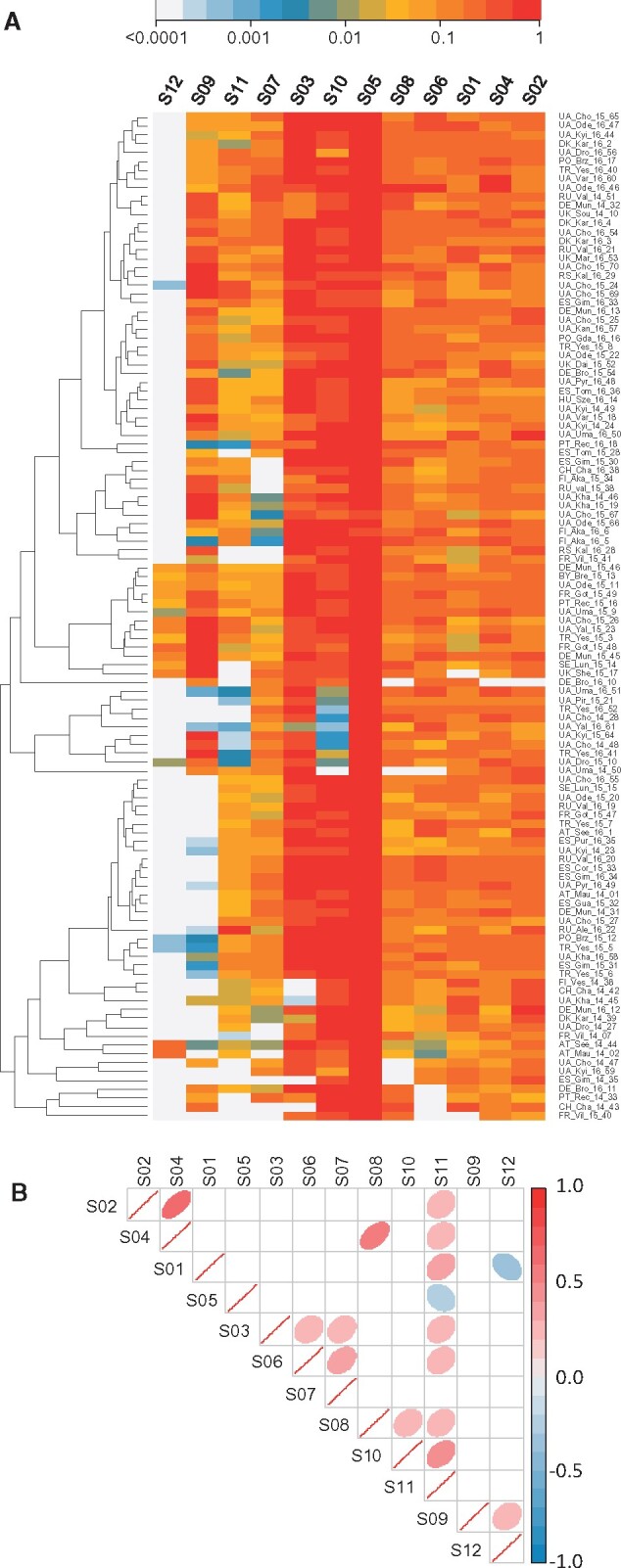
Drosophila Vesanto virus segment copy-number. (A) Heatmap showing the relative number of sequencing reads from each of the twelve Vesanto virus segments (columns), for each of the population samples (rows). Populations are included if at least one segment appeared at 1 per cent of the fly genome copy-number. Rows and columns have been ordered by similarity (dendrogram) to identify structure within the data. Colours show copy-number relative to the highest-copy segment, on a log scale. (B) Correlations in copy-number among the segments, with ‘significant’ correlations (*P* < 0.05, no corrections) shown with coloured ellipses, according to the direction (red positive, blue negative) and strength of correlation. The absence of strong negative correlations between segments encoding homologous proteins (e.g. S01, S03, S11, which all encode genes with homology to DNA Polymerase B) may indicate that these segments do not substitute for each other.

While examining an assembly graph of sample UA_Kan_16_57, we noted a third scaffold with a similarly high coverage (>300-fold) and similar structure (4.8 kb in length with inverted terminal repeats). This sequence also appeared to encode a protein with distant homology to bidnavirus DNA polymerase B, and we reasoned that it might represent an additional virus. We therefore mapped reads from datasets that had high coverage of Drosophila Vesanto virus segments S01 and S02 to all scaffolds from the *de novo* build of UA_Kan_16_57, with the objective of finding any additional segments based on their co-occurrence across datasets (e.g. as done by Batson et al. 2020; Obbard et al. 2020). This identified several possible segments, all between 3.3 and 5.8 kbp in length and possessing inverted terminal repeats. We then used their translated open reading frames to search all of our *de novo* builds, and in this way identified a total of twelve distinct segments that show structural similarity and a strong pattern of co-occurrence (Figs 1 and 3; figshare repository 10.6084/m9.figshare.14161250 S5). To capture the diversity present among these putative viruses, we made targeted *de novo* builds of three datasets, incorporating both Illumina reads and Oxford nanopore reads (Table 1). We have submitted these contigs to GenBank as MT496850–MT496878, and additional sequences are provided in figshare repository 10.6084/m9.figshare.14161250 S6. Because the inverted terminal repeats and pooled sequencing of multiple infections make such an assembly particularly challenging, we also sought to support these structures by identifying individual corroborating Nanopore reads of 2 kbp or more. We believe the inverted terminal repeat sequences should be treated with caution, but it is nevertheless striking that many of these putative segments show sequence similarity in their terminal inverted repeats, as commonly seen for segmented viruses.

Although we identified twelve distinct segments with strongly correlated presence/absence, not all segments were detectable in all affected samples (Fig. 3A). Only segment S05, which encodes a putative glycoprotein and a putative nuclease domain protein, was always detectable in samples containing *Drosophila* Vesanto virus (in ninety-one of the 167 samples; figshare repository 10.6084/m9.figshare.14161250 S5). Several segments were very commonly detectable, such as S03 (protein with homology to DNA PolB) and S10 (encoding a protein with domain of unknown function DUF3472 and a putative glycoprotein) in around seventy samples, and segments S01, S02, S04, S06, and S08 in around fifty-five samples. Others were extremely rare, such as S12 (encoding a putative NACHT domain protein with homology to S09), which was only seen in five samples. We considered three possible explanations for this pattern.

Our first hypothesis was that Drosophila Vesanto virus has twelve segments, but that variable copy number among the segments causes some to occasionally drop below the detection threshold. In support of this, all segments are indeed detectable in the sample with the highest Drosophila Vesanto virus read numbers (FR_Got_15_49), ranging from seven-fold higher than the fly genome for S07 to 137-fold higher for S05. In addition, ‘universal’ segment S05 is not only the most widely detected segment across samples, but also has the highest average read depth within samples. However, despite 1.6 million Drosophila Vesanto virus reads in the second highest copy-number sample (RU_Val_16_20; 125-fold more copies of S06 than of *Drosophila*), no reads mapped to S12, strongly suggesting the absence of S12 from this sample. Our second hypothesis was that some segments are optional or satellite segments, or may represent alternative versions of other, homologous segments, comprising a re-assorting community (as in influenza viruses). The latter is consistent with the apparent homology between some segments. For example, S01, S03, and S11 all encode DNA Polymerase B-homologs, and S06, S07, and S10 all encode DUF3472 proteins. It is also consistent with the universal presence of S05, which appears to lack homologs. However, two of the DNA PolB homologs are highly divergent (Fig. 2C) to the extent it is hard to be confident of polymerase function, and we could not detect compelling negative correlations between homologous segments that might suggest that they substitute for each other in different populations (Fig. 3B). Our third hypothesis was that ‘Drosophila Vesanto virus’ in fact represents multiple independent viruses (or phage), and that the superficially clear pattern of co-occurrence is driven by high (hypothetical) prevalence of this virus community in an occasional member of the *Drosophila* microbiota, such as a fungus or trypanosomatid. However, we were unable to detect any correlation with the mapped microbiota reads, and high levels of Drosophila Vesanto virus are seen in samples with few un-attributable reads. For example, sample PO_Brz_15_12 has eleven-fold more copies of S06 than of the fly genome, but <2 per cent of reads derive from an unknown source (figshare repository 10.6084/m9.figshare.14161250 S3).

### 3.4 The complete genome of a new divergent entomopoxvirus

Kapun *et al.* (2020) also reported the presence of a pox-like virus in DrosEU data from 2014, but were unable to assemble the genome. By incorporating a small number of long sequencing reads, and using targeted reassembly combined with manual examination of the assembly graph, we were able to assemble this genome from dataset UA_Yal_14_16 (SRR5647764) into a single contig of 219.9 kb. As expected for pox-like viruses, the genome appears to be linear with long inverted terminal repeats of 8.4 kb, and outside of the inverted terminal repeats sequencing coverage was 15.7-fold (Fig. 1). We refer to this virus as ‘Drosophila Yalta entomopoxvirus’, reflecting the collection location (Yalta, Ukraine), and we have submitted the sequence to GenBank under accession number MT364305. This virus has very recently been shown to be most closely related to Diachasmimorpha longicaudata entomopoxvirus (Coffman and Burke 2020).

Within the Drosophila Yalta entomopoxvirus genome we identified a total of 177 predicted proteins, including forty-six of the forty-nine core poxvirus genes, and missing only the E6R virion protein, the D4R uracil-DNA glycosylase, and the 35 kDa RNA polymerase subunit A29L (Upton et al. 2003). Interestingly, the genome has a higher GC content than the other previously published entomopoxviruses, which as a group consistently display the lowest GC content (<21%) of the poxviruses (Perera et al. 2010; Thézé et al. 2013). Consistent with this, our phylogenetic analysis of three concatenated protein sequences suggests that the virus is distantly related, falling only slightly closer to entomopoxviruses than other poxviruses (Fig. 2D). Given that all poxviruses infect metazoa, and that no animal species other than *D.melanogaster* appeared to be present in the sample, we believe *D.melanogaster* is likely to be the host.

### 3.5 Two new complete nudivirus genomes, and evidence for a third

In addition to Drosophila Kallithea nudivirus and Drosophila Esparto nudivirus, our expanded analysis identified three novel nudiviruses that were absent from data collected in 2014. We were able to assemble two of these into complete circular genomes of 112.3 kb (twenty-seven-fold coverage) and 154.5 kb (forty-one-fold coverage), respectively, based on datasets from Tomelloso, Spain (ES_Tom_15_28; SRR8439136) and Mauternbach, Austria (AT_Mau_15_50; SRR8439127). We refer to these viruses as ‘Drosophila Tomelloso nudivirus’ and ‘Drosophila Mauternbach nudivirus’, reflecting the collection locations, and we have submitted the sequences to GenBank under accession numbers KY457233 and MG969167. We predict Drosophila Tomelloso nudivirus to encode 133 proteins (Fig. 1), and phylogenetic analysis suggests that it is more closely related to a beetle virus (Oryctes rhinocerous nudivirus, Fig. 2A; Etebari et al. 2020) than to the other nudiviruses described from *Drosophila*. Drosophila Mauternbach nudivirus is predicted to encode ninety-five proteins (Fig. 1), and is very closely related to Drosophila innubila nudivirus (Fig. 2A; Unckless 2011; Hill and Unckless 2018). However, synonymous divergence (*K_S_*) between these two viruses is ∼0.7 that is, nearly six-fold more than that between *D.melanogaster* and *D.simulans*, supporting their consideration as distinct ‘species’. The third novel nudivirus was present at a very low level in a sample from Kaniv, Ukraine (UA_Kan_16_57, SRR8494448), and only small fragments of the virus could be assembled for phylogenetic analysis (GenBank accession MT496841-MT496846). This showed that the fragmentary nudivirus from Kaniv is approximately equally divergent from Drosophila innubila nudivirus and Drosophila Mauternbach nudivirus (Fig. 2A).

The collections from Tomelloso and Kaniv did not contain reads mapping to *Drosophila* species other than *D.melanogaster*, or to nematode worms or mites. Moreover, we identified Drosophila Tomelloso nudivirus in a number of experimental laboratory datasets from *D.melanogaster* (see below; Riddiford et al. 2020), and these lacked a substantial microbiome. Together these observations strongly support *D.melanogaster* as a host for these viruses. In contrast, *COI* reads suggest that the sample from Mauternbach may have contained one *D.phalerata* individual (2.4% of diagnostic nuclear reads; figshare repository 10.6084/m9.figshare.14161250 S3). And, as we could not detect Drosophila Mauternbach nudivirus in any of the public datasets we examined (below), we remain uncertain whether *D.melanogaster* or *D.phalerata* was the true host.

### 3.6 Evidence for a new filamentous virus and a new hytrosavirus

Our search also identified fragments of two further large dsDNA viruses from lineages that have not previously been reported to naturally infect Drosophilidae. First, in sample UA_Ode_16_47 (SRR8494427) from Odesa, Ukraine, we identified around 16.6 kb of a novel virus related to the salivary gland hypertrophy viruses of *Musca domestica* and *Glossina palpides* (Fig. 2A; Prompiboon et al. 2010; Kariithi et al. 2013). Our assembled fragments comprised eighteen short contigs of only one- to three-fold coverage (submitted under accessions MT469997–MT470014). As the *Glossina* and *Musca* viruses have circular dsDNA genomes of 124.3 and 190.2 kbp, respectively, we believe that we have likely sequenced 5–15 per cent of the genome. Because this population sample contains a small number of reads from *D.simulans* and an unknown nematode worm related to *P.redivivus*, and because we were unable to detect this virus in public datasets from *D.melanogaster* (below), the true host remains uncertain. However, given that the closest relatives all infect Diptera, it seems likely that either *D.melanogaster* or *D.simulans* is the host.

Second, in sample ES_Gim_15_30 (SRR8439138) from Gimenells, Spain, we identified around 86.5 kb of a novel virus distantly related to the filamentous virus of *L.boulardi*, a parasitoid wasp that commonly attacks *Drosophila* (Fig. 2; Lepetit et al. 2016). The assembled fragments comprised nine scaffolds of 5.9–16.9 kbp in length and three- to ten-fold coverage, and are predicted to encode sixty-nine proteins (scaffolds submitted to GenBank under accessions MT496832–MT496840). Leptopilina boulardi filamentous virus has a circular genome of 111.5 kbp predicted to encode 108 proteins. This suggests that, although fragmentary, our assembly may represent most of the virus. A small number of reads from ES_Gim_15_30 mapped to a relative of nematode *P.redivivus* and, surprisingly, to the Atlantic salmon (*Salmo salar*), but we consider these unlikely hosts as the level of contamination was very low and other filamentous viruses are known to infect insects. We were unable to detect the novel filamentous virus in any public datasets from *D.melanogaster* (below), and given that Leptopilina boulardi filamentous virus infects a parasitoid of *Drosophila*, it is possible that this virus may similarly infect a parasitoid wasp rather than the fly. However, as we were unable to detect any reads mapping to *Leptopilina* or other parasitoids of *Drosophila* in any of our samples, we think *D.melanogaster* is a good candidate to be a true host.

### 3.7 Near-complete genomes of three adintoviruses

Based on the presence of a capsid protein, it is thought that some Polinton-like transposable elements (also known as Mavericks) are actually horizontally transmitted viruses (Yutin et al. 2015). Some of these have recently been proposed as the *Adintoviridae*, a family of dsDNA viruses related to bidnaviruses and other PolB-encoding DNA viruses (Starrett et al. 2020). We identified three possible adintoviruses in DrosEU data. The first, which we refer to as Drosophila-associated adintovirus 1, occurred in sample UA_Cho_15_26 (SRR8439134) from Kopachi (Chornobyl Exclusion Zone), Ukraine and comprised a single contig of 14.5 kb predicted to encode twelve proteins. Among these proteins are not only a DNA Polymerase B and an integrase, but also homologs of the putative capsid, virion-maturation protease, and FtsK proteins of adintoviruses (Starrett et al. 2020), and possibly very distant homologs of hytrosavirus gene MdSGHV056 and ichnovirus gene AsIV-cont00038 (Fig. 1). The second, which we refer to as Drosophila-associated adintovirus 2, is represented by a 13.3 kb contig assembled using AT_Mau_15_50 from Mauternbach, Austria (SRR8439127). It is very closely related to the first adintovirus, and encodes an almost-identical complement of proteins (Fig. 1). In a phylogenetic analysis of DNA PolB sequences, both fall close to sequences annotated as polintons in other species of *Drosophila* (Fig. 2C). It is notable that these two datasets are those that are contaminated by *D.testacea* and *D.phalerata*, respectively. We therefore think it likely that Drosophila-associated adintovirus 1 and 2 are associated with those two species rather than *D.melanogaster*, and may potentially be integrated into their genomes. These sequences have been submitted to GenBank under accessions MT496847 and MT496848.

In contrast, Drosophila-associated adintovirus 3 was assembled using sample DK_Kar_16_4 from Karensminde, Denmark (SRR8494437), from which other members of the Drosophilidae were absent. It is similarly 13.8 kb long, and our phylogenetic analysis of DNA PolB places it within the published diversity of insect adintoviruses—although divergent from other adintoviruses or polintons of *Drosophila* (Fig. 2C; see also Starrett et al. 2020). However, this sequence is only predicted to encode ten proteins and these are generally more divergent, perhaps suggesting that this virus is associated with a completely different host species, such as the nematode related to *P.redivivus* or a trypanosomatid—although these species were present at very low levels. The sequence has been submitted to GenBank under accession MT496849.

### 3.8 Prevalence varies among viruses, and in space and time

**Figure 4.**
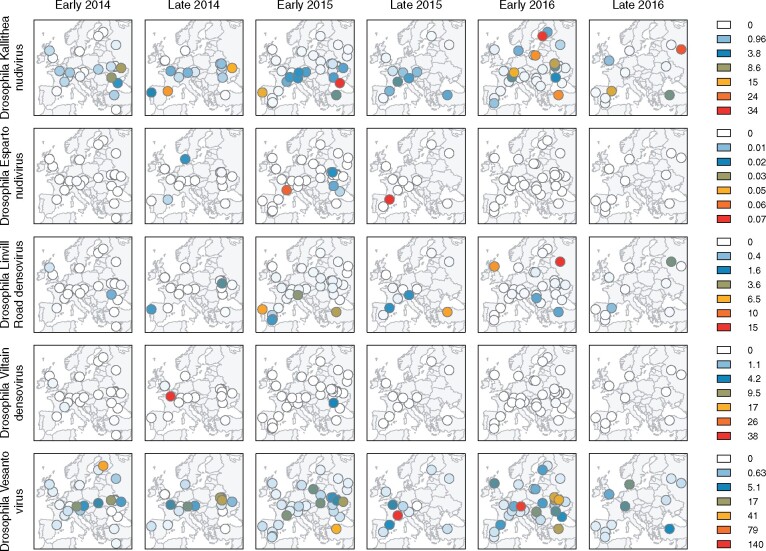
Geographic distribution of DNA virus reads in European *D.melanogaster*. Maps show the spatial distribution of virus read copy-number (relative to fly genomes) on a non-linear colour scale. Data are shown for the five viruses that were each detected more than once (rows), separated by year and whether flies were collected relatively ‘early’ or ‘late’ in the season (columns).

Based on a detection threshold of 1 per cent of the *Drosophila* genome copy-number, only five of the viruses (Drosophila Kallithea nudivirus, Drosophila Vesanto virus, Drosophila Linvill Road densovirus, Drosophila Viltain densovirus, and Drosophila Esparto nudivirus) were detectable in multiple population pools. The other nine viruses were each detectable in a single pool. For viruses in a single pool, a simple maximum-likelihood estimate of prevalence—assuming independence of flies and pools—is 0.015 per cent (with an upper 2-log likelihood bound of 0.07 per cent). Among the intermediate-prevalence viruses, Drosophila Esparto nudivirus and Drosophila Viltain virus were detected in five pools each, corresponding to a prevalence of 0.08 per cent (0.03–0.17%), and Drosophila Linvill road densovirus was detected in twenty-one pools, indicating a prevalence of 0.34 per cent (0.21–0.51%). The two most common viruses were Drosophila Kallithea nudivirus, which was detected in ninety-three pools giving a prevalence estimate of 2.1 per cent (1.6–2.5%), and Drosophila Vesanto virus, which was detected in 114 pools giving a prevalence estimate of 2.9 per cent (2.4–3.5%). However, it should be noted that all flies were male, and if virus prevalence differs between males and females then these estimates could be misleading. Both virulence and titre can differ between the sexes (e.g. in Drosophila Kallithea nudivirus; Palmer et al. 2018), although differences in prevalence were not found for RNA viruses of *Drosophila* (Webster et al. 2015; Fig. 4).

**Figure 5.**
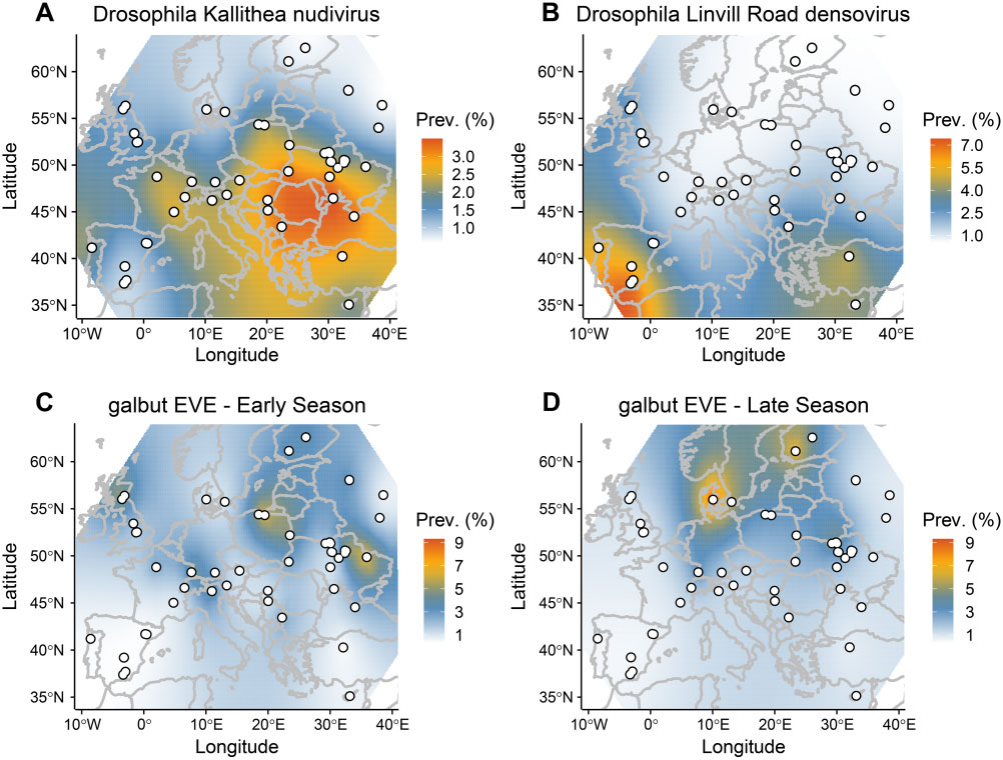
Geographic variation in estimated prevalence: Drosophila Kallithea nudivirus (A), Drosophila Linvill Road denosovirus (B), and the galbut virus EVE (C and D). Sampling sites are marked as white dots, and the colour gradient illustrates predictions from the INLA model, but with scale transformed to the predicted individual-level prevalence (%), assuming independence among individuals and population samples of size 40. Only Drosophila Kallithea nudivirus, Drosophila Linvill Road densovirus, and the galbut virus EVE displayed a significant spatial component, and only the EVE differed between seasons.

Drosophila Kallithea nudivirus, Drosophila Vesanto virus, and Drosophila Linvill Road densovirus were sufficiently prevalent to analyse their presence/absence across populations using a Bayesian spatial generalised linear mixed model. Our analysis identified a spatial component to the distribution of both Drosophila Kallithea nudivirus and Drosophila Linvill Road densovirus that did not differ significantly between years, with a higher prevalence of Drosophila Kallithea nudivirus in southern and central Europe, and a higher prevalence of Drosophila Linvill Road densovirus in Iberia (Fig. 5A and B; ΔDIC of −13.6 and −17.2, respectively, explaining 15.5 and 32.8 per cent of the variance). In contrast, Drosophila Vesanto virus showed no detectable spatial variation in prevalence, but did vary significantly over time, with a significantly lower prevalence in 2014 compared with the other years (2015 and 2016 were higher by 1.27 [0.42, 2.16] and 1.43 [0.50, 2.14] respectively). The probability of observing a virus did not depend on the sampling season or the amount of *Wolbachia* in the sample.

As sampling location did not explain any significant variation in the probability of detecting any virus, it appears that—beyond broad geographic trends—there is little temporal consistency in virus prevalence at the small scale. At the broader geographic scale, it seems likely that climatic factors, directly or indirectly, play a role; it may be that temperature and humidity affect virus transmission, as seen for many human viruses (Moriyama, Hugentobler, and Iwasaki 2020). Equally, host density and demography are strongly affected by climate, and will affect the opportunity for transmission, both within and between host species. For example, the probability of detecting Drosophila Linvill Road densovirus was positively correlated with the level of *D.simulans* contamination (95% credible interval of the log-odds ratio [2.9, 14.6]), suggesting either that some reads derived from infections of *D.simulans* (in which the virus can have very high prevalence, see data from Signor, New, and Nuzhdin 2018), or that infections in *D.melanogaster* may be associated with spillover from *D.simulans*.

### 3.9 DNA viruses are detectable in publicly available *Drosophila* datasets

We wished to corroborate our claim that these viruses are associated with *Drosophila* by exploring their prevalence in laboratory populations and publicly available data. We therefore examined the first 10 million reads from each of 3,003 sequencing runs from 28 *D.melanogaster* and *D.simulans* sequencing projects. In general, our survey suggests that studies using isofemale or inbred laboratory lines tend to lack DNA viruses (e.g. Mackay et al. 2012; Grenier et al. 2015; Lack et al. 2015; Gilks et al. 2016; Lange et al. 2016). In contrast, studies that used wild-caught or F1 flies (e.g. Endler et al. 2018; Machado et al. 2019) or large population cages (e.g. Schou et al. 2017) were more likely to retain DNA viruses (figshare repository 10.6084/m9.figshare.14161250 S7).

Based on our detection thresholds, none of the public datasets we examined appeared to contain Drosophila Mauternbach nudivirus, Drosophila Yalta entomopoxvirus, the filamentous virus, the hytrosavirus, or the three adintoviruses (figshare repository 10.6084/m9.figshare.14161250 S7). This is consistent with their extreme rarity in our own sampling, and the possibility that Drosophila Mauternbach nudivirus and the adintoviruses may actually infect species other than *D.melanogaster*. Although some reads from Dros-RTEC run SRR3939056 (ninety-nine flies from Athens, Georgia; Machado et al. 2019) did map to an adintovirus, these reads actually derive from a distinct virus that has only 82 per cent nucleotide identity to *Drosophila*-associated adintovirus-1. Unfortunately, this closely related adintovirus cannot corroborate the presence of *Drosophila*-associated adintovirus-1 in *D.melanogaster*, as run SRR3939056 is contaminated with *Scaptodrosophila latifasciaeformis*, which could be the host.

One of our rare viruses was present (but also rare) in public data: Drosophila Viltain densovirus appeared only once in 3,003 sequencing datasets, in one of the sixty-three libraries from Dros-RTEC project PRJNA308584 (Machado et al. 2019). Drosophila Tomelloso nudivirus, which was rare in our data, was more common in public data, appearing in five of twenty-eight projects and twenty-three of 3,003 runs. However, this may explained by its presence in multiple runs from each of a small number of experimental studies (e.g., Fang et al. 2017; Liu and Secombe 2015; Riddiford et al. 2020; Siudeja et al. 2015). Our three most common viruses were also the most common DNA viruses in public data. Drosophila Linvill Road densovirus appeared in ten of the twenty-eight projects we examined, including 363 of the 3,003 runs. This virus was an exception to the general rule that DNA viruses tend to be absent from inbred or long-term laboratory lines, as it was detectable in 166 of 183 sequencing runs of inbred *D.simulans* (Signor, New, and Nuzhdin 2018). Drosophila Kallithea nudivirus appeared in four of the twenty-eight projects, including sixty of the runs, and was detectable in wild collections of both *D.melanogaster* and *D.simulans*. Drosophila Vesanto virus was detectable in eight of the twenty-eight projects, including 208 of the runs, but only in *D.melanogaster* datasets.

The presence of Drosophila Vesanto virus segments in public data is of particular value because it could help to elucidate patterns of segment co-occurrence. This virus was highly prevalent in a large experimental evolution study using caged populations of *D.melanogaster* derived from collections in Denmark in 2010 (Schou et al. 2017), where segments S01, S02, S04, S05, and S10 were almost always present, S03, S06, S07, and S08 were variable, and S09, S11, and S12 were always absent. However, because these data were derived from restriction associated digest sequencing, absences may reflect absence of the restriction sites. Drosophila Vesanto virus also appeared in pooled GWAS datasets (e.g. Endler et al. 2018), for which segments S09 and S12 were always absent and segments S03, S10, and S11 were variable (figshare repository 10.6084/m9.figshare.14161250 S7), and in several Dros-RTEC datasets (Machado et al. 2019) in which only S12 was consistently absent. Unfortunately, it is difficult to test among the competing hypotheses using pooled sequencing of wild-collected flies or large cage cultures. This is because different flies in the pool may be infected with different viruses or with viruses that have a different segment composition, and because a more complex microbiome may be present. However, we were able to find one dataset from an isofemale line, GA10 collected in Athens, Georgia (USA) in 2009, that had been maintained in the laboratory for at least five generations prior to sequencing (ERR705977 from Bergman and Haddrill 2015). From this dataset we assembled eight of the twelve segments, including two segments encoding PolB-like proteins and two encoding the DUF3472 protein. Mapping identified no reads at all from segments S9 or S12. This most strongly supports a single virus with a variable segment composition between infections and/or re-assortment. Moreover, the low species complexity of this laboratory dataset supports *D.melanogaster* as the host, with over 98 per cent of reads mapped, and with *Drosophila*, *Wolbachia*, and *Lactobacillus plantarum* the only taxa present in appreciable amounts. Example Vesanto virus sequences from these datasets are provided in figshare repository 10.6084/m9.figshare.14161250 S6.

### 3.10 Genetic diversity varies among viruses and populations

We examined genetic variation in three of the most common viruses; Drosophila Kallithea nudivirus, Drosophila Linvill Road densovirus, and Drosophila Vesanto virus. After masking regions containing indels, and using a 1 per cent MAF threshold for inclusion, we identified 923 SNPs across the total global Drosophila Kallithea nudivirus pool, and 15,132 distinct SNPs summed across the forty-four population samples. Of these SNPs, 13,291 were private to a single population, suggesting that the vast majority of Drosophila Kallithea nudivirus SNPs are globally and locally rare and limited to one or a few populations. This is consistent with many of the variants being recent and/or deleterious, but could also reflect a large proportion of sequencing errors—despite the analysis requiring a MAF of 1 per cent and high base quality. Synonymous pairwise genetic diversity in the global pool was very low, at π_S_ = 0.15 per cent, with π at intergenic sites being almost identical (0.14%). Diversity did not vary systematically around the virus genome (figshare repository 10.6084/m9.figshare.14161250 S9). Consistent with the large number of low-frequency private SNPs, average within population-pool diversity was ten-fold lower still, at π_S_ = 0.04 per cent, corresponding to a very high *F_ST_* of 0.71. In general, the level of constraint on virus genes appeared low, with global π_A_/π_S_ 0.39 and local π_A_/π_S_ = 0.58. These patterns of diversity are markedly different to those of the host, in which π_S_ (at four-fold degenerate sites) is on the order of 1 per cent with π_A_/π_S_ (zero- and four-fold) around 0.2, and differentiation approximately *F_ST_* = 0.03 (Kapun et al. 2020; Tristan, Aurelie, and Annabelle 2019). Given that large dsDNA virus mutation rates can be ten- to 100-fold higher than animal mutation rates (Duffy 2018), the overall lower diversity in Drosophila Kallithea nudivirus is consistent with bottlenecks during infection and the smaller population size that corresponds to a 2.1 per cent prevalence. The very low within-population diversity and high *F_ST_* and π_A_/π_S_ may be indicative of local epidemics, or a small number of infected hosts within each pool (expected to be 1.47 infected flies in an infected pool, assuming independence) with relatively weak constraint. Alternatively, high *F_ST_* and π_A_/π_S_ may indicate a high proportion of sequencing errors.

In Drosophila Vesanto virus we identified 4,059 SNPs across all segments and divergent segment haplotypes in the global pool, with 5,491 distinct SNPs summed across all infected population samples, of which 4,235 were private to a single population. This corresponded to global and local diversity that was around seven-fold higher than Drosophila Kallithea nudivirus (global π_S_ = 1.16%, local π_S_ = 0.28%), and to intermediate levels of constraint on the protein sequence (π_A_/π_S_ = 0.20), but a similar level of differentiation (*F_ST_* = 0.76). Although the prevalence of Drosophila Vesanto virus appears to be slightly higher than Drosophila Kallithea nudivirus (2.9% vs. 2.1%), much of the difference in diversity is probably attributable to the higher mutation rates of ssDNA viruses (Duffy 2018). The apparent difference in the allele frequency distribution between these two viruses is harder to explain (73% of SNPs detectable at a global MAF of 1%, vs. only 6% in Drosophila Kallithea nudivirus), but could be the result of the very strong constraint on protein coding sequences keeping non-synonymous variants below the 1 per cent MAF threshold even within local populations. It is worth noting that the difference between Drosophila Vesanto virus and Drosophila Kallithea nudivirus in π_A_/π_S_ and the frequency of rare alleles argues against their being purely a result of sequencing error in Drosophila Kallithea nudivirus, as the error rates would be expected to be similar between the two viruses,

In Drosophila Linvill Road densovirus, which was only present in thirteen populations and has the smallest genome, we identified 178 SNPs across the global pool, and 253 distinct SNPs summed across the infected populations, of which 209 were private to a single population. Although this virus appears at least six-fold less prevalent than Drosophila Kallithea nudivirus or Drosophila Vesanto virus, it displayed relatively high levels of genetic diversity both globally and locally (global π_S_ = 1.45%, local π_S_ = 0.21%, *F_ST_* = 0.86), and higher levels of constraint on the protein sequence (π_A_/π_S_ = 0.10). Given a mutation rate that is likely to be similar to that of Drosophila Vesanto virus, this is hard to reconcile with a prevalence that is six-fold lower. However, one likely explanation is that Drosophila Linvill Road densovirus is more prevalent in the sister species *D.simulans* (above), and the diversity seen here represents rare spillover and contamination of some samples with that species.

### 3.11 Structural variation and TEs in Drosophila Kallithea Nudivirus

*De novo* assembly of Drosophila Kallithea nudivirus from each sample resulted in fifty-two populations with complete single-scaffold genomes that ranged in length from 151.7 to 155.9 kbp. Alignment showed these population-consensus assemblies to be co-linear with a few short duplications of 10–100 nt, but generally little large-scale duplication or rearrangement. Two regions were an exception to this: that spanning positions 152,180–152,263 in the circular reference genome (between putative proteins AQN78547 and AQN78553; genome KX130344.1), and that spanning 67,903 to 68,513 (within putative protein AQN78615). The first region comprised multiple repeats of around 100 nt and assembled with lengths ranging from 0.2 to 3.6 kbp, and the second comprised multiple repeats of around 140 nt and assembled with lengths between 0.5 and 2.4 kbp. Together, these regions explained the majority of the length variation among the Kallithea virus genome assemblies. We also sought to catalogue small-scale indel variation in Kallithea virus by analysing indels within reads. In total, after indel-realignment using GATK, across all forty-four infected samples we identified 2,289 indel positions in the Drosophila Kallithea nudivirus genome that were supported by at least five reads. However, only 195 of these indels were at high frequency (over 50% of samples). As would be expected, the majority (1,774) were found in intergenic regions (figshare repository 10.6084/m9.figshare.14161250 S9).

Pooled assemblies can identify structural variants that differ in frequency among populations, but they are unlikely to identify rare variants within populations, such as those caused by TE insertions. TEs are commonly inserted into large DNA viruses, and these viruses have been proposed as a vector for interspecies transmission of TEs (Gilbert et al. 2016; Gilbert and Cordaux 2017). In total, we identified 5,169 read pairs (across sixteen datasets with >300-fold coverage of Drosophila Kallithea nudivirus) that aligned to both *D.melanogaster* TEs and Drosophila Kallithea nudivirus. However, the vast majority of these (5,124 out of 5,169) aligned internally to TEs, more than 5 bp away from the start or end position of the TE, which is inconsistent with insertion (Gilbert et al. 2016; Loiseau et al. 2020). Instead, this pattern suggests PCR-mediated recombination, and assuming that all chimaeras we found were artefactual, their proportion among all reads mapping to the Kallithea virus (0.01%) falls in the lower range of that found in other studies (Peccoud et al. 2018). We therefore believe there is no evidence supporting *bona fide* transposition of *D.melanogaster* TEs into genomes of the Drosophila Kallithea nudivirus in these natural virus isolates. This is in striking contrast to what was found in the Autographa californica multiple nucleopolyhedrovirus (Loiseau et al. 2020) and could perhaps reflect the tropism of Drosophila Kallithea nudivirus (Palmer et al. 2018) to tissues that experience low levels of transposition.

### 3.12 A genomic insertion of galbut virus is segregating in *D.melanogaster*

The only RNA virus we identified among the DNA reads from DrosEU collections was galbut virus, a segmented and bi-parentally vertically transmitted dsRNA partitivirus that is extremely common in *D.melanogaster* and *D.simulans* (Webster et al. 2015; Cross et al. 2020). Based on a detection threshold of 0.1 per cent of fly genome copy number, galbut virus reads were present in forty-three out of 167 samples. There are two likely sources of such DNA reads from an RNA virus in *Drosophila*. First, reads might derive from somatic circular DNA copies that are reported to occur as a part of the immune response (Mondotte et al. 2018; Poirier et al. 2018). Second, reads might derive from a germline genomic integration that is segregating in wild populations (i.e. an EVE; Katzourakis and Gifford 2010; Tassetto et al. 2019). We sought to distinguish between these possibilities by *de novo* assembly of the galbut sequences from high copy-number DrosEU samples and public *D.melanogaster* DNA datasets.

**Figure 6.**
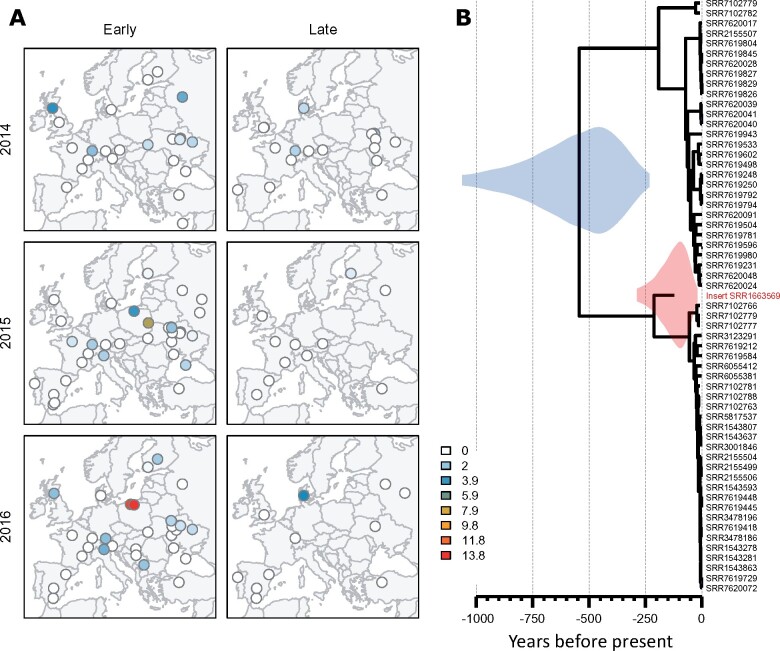
*Drosophila melanogaster* harbours an endogenous genomic copy of galbut virus. (A) Maps show the spatial distribution of the DNA reads from the galbut EVE, as a percentage of fly genomes (maximum 13.8%) on colour scale. Rows show years of sampling, and columns show ‘early’ or ‘late’ samples in each year (B) The relationship between the galbut EVE and galbut virus sequences detectable in public datasets, illustrated by a Bayesian maximum clade-credibility tree inferred under a strict clock, with median-scaled node dates. The 95 per cent highest posterior density for the root date of extant galbut viruses is shown in blue (230–1,060 years before present), and the 95 per cent highest posterior density for the inferred date of insertion, is shown in red (20–290 years before present).

We assembled the galbut virus sequence from the three DrosEU samples in which it occurred at high read-depth: BY_Bre_15_13 (Brest, Belarus), PO_Gda_16_16 (Gdansk, Poland), and PO_Brz_16_17 (Brzezina, Poland). We were also able to assemble the sequence from four publicly available sequencing runs: three (SRR088715, SRR098913, and SRR1663569) that we believe are derived from global diversity line N14 (Grenier et al. 2015) collected in The Netherlands in 2002 (Bochdanovits and de Jong 2003), and SRR5762793, which was collected in Italy in 2011 (Mateo, Rech, and González 2018). In every case, the assembled sequence was an identical 1.68 kb, near full-length, copy of galbut virus segment S03, including the whole of the coding sequence for the viral RNA-dependent RNA polymerase. Also, in every case, this sequence was inserted into the same location in a 297 Gypsy-like LTR retrotransposon (i.e. identical breakpoints), around 400 bp from the 5′ end. This strongly suggests that these galbut sequences represent a unique germline insertion: Even if the insertion site used in the immune response were constant, the inserted virus sequence would be highly variable across Europe over 14 years. The sequence falls among extant galbut virus sequences (Fig. 6B), and is 5 per cent divergent (18.5% synonymous divergence) from the closest one available in public data. The sequences are provided in figshare repository 10.6084/m9.figshare.14161250 S10

Interestingly, populations with a substantial number of galbut virus reads (a maximum of 13.8% or 11 chromosomes of 80) appeared geographically limited, appearing more commonly in higher latitudes, and with a different spatial distribution in the early and late collecting seasons (ΔDIC = 26.92; Figs 5C and 6A). Given the absence of this sequence from Dros-RTEC (Machado et al. 2019), Drosophila Genetic Reference Panel (Mackay et al. 2012), and the other *Drosophila* Genome Nexus datasets (Lack et al. 2015; Lange et al. 2016), it seems likely that this insertion is of a recent, likely northern or central European, origin. We used a strict-clock phylogenetic analysis of viral sequences to estimate that the insertion occurred within the last 300 years (posterior mean 138 years ago, 95% highest posterior density interval 20–287 years ago; Fig. 6B) that is, after *D.melanogaster* was spreading within Europe. Unfortunately, the insertion site in a high copy-number TE means that we were unable to locate it in the genome. This also means that it was not possible to detect whether the insertion falls within a piwi-interacting RNA (piRNA)-generating locus, which is seen for several EVEs in mosquitoes (Palatini et al. 2017) and could perhaps provide resistance to the vertically transmitted virus. Surprisingly, DNA reads from galbut virus were more likely to be detected at sites with a higher percentage of reads mapping to *Wolbachia* (95% credible interval for the effect [0.074, 0.41]; ΔDIC = −5.52). Given that no correlation between galbut virus and *Wolbachia* has been detected in the wild (Shi et al. 2018b; Webster et al. 2015), we think this most likely reflects a chance association between the geographic origin of the insertion and the spatial distribution of *Wolbachia* loads (Kapun et al. 2020).

## 4. Discussion

Although metagenomic studies are routinely used to identify viruses and virus-like sequences (e.g. Shi, Zhang, and Holmes 2018a; Zhang, Shi, and Holmes 2018), simple bulk sequencing can only show the presence of viral sequences; it cannot show that the virus is replicating or transmissible, nor can it unequivocally identify the host (reviewed in Obbard 2018). This behoves metagenomic studies to carefully consider any additional evidence that might add to, or detract from, the claim that an ‘associated virus-like sequence’ is indeed a virus. A couple of the DNA viruses described here undoubtedly infect *Drosophila*. Drosophila Kallithea nudivirus has been isolated and studied experimentally (Palmer et al. 2018), and Drosophila Tomelloso nudivirus is detectable in some long-term laboratory cultures (e.g. Liu and Secombe 2015; Fang et al. 2017; Siudeja et al. 2015; Riddiford et al. 2020). Others, such as Drosophila Viltain densovirus, Drosophila Linvill Road densovirus, and Drosophila Vesanto virus, are present at such high copy numbers, and sometimes in laboratory cultures, that any host other than *Drosophila* seems very unlikely. Some, appearing at reasonable copy number but in a single sample, could be infections of contaminating *Drosophila* species (Drosophila Mauternbach nudivirus, the adintoviruses), or spillover from infections of parasitoid wasps (Drosophila Yalta entomopoxvirus, the filamentous virus). A few, having appeared at low copy number in a single sample, could be contaminants—although we excluded virus-like sequences that appeared strongly associated with contaminating taxa (figshare repository 10.6084/m9.figshare.14161250 S4).

These caveats aside, along with Drosophila innubila nudivirus (Unckless 2011) and IIV31 in *D.obscura* and *D.immigrans* (Webster et al. 2016), our study increases the total number of published DNA viruses associated with *Drosophila* to sixteen. Although a small sample, these viruses hint at some interesting natural history. First, it is striking that more than a third of the reported DNA viruses are nudiviruses (six of the sixteen published, plus a seventh from *Phortica variegata*; Fig. 2). This suggests that members of the *Nudiviridae* are common pathogens of *Drosophila*, and may indicate long-term host lineage fidelity with short-term switching among species. Such switching is consistent with the apparent lack of congruence between host and virus phylogenies, and the fact that both Drosophila innubila nudivirus and Drosophila Kallithea nudivirus infect multiple *Drosophila* species (Fig. 2). Second, the majority of DNA viruses seem to be rare. Seven of the twelve viruses confidently ascribable to *D.melanogaster* or *D.simulans* were detected in just one of the 167 population samples, and likely only one of 6,668 flies, consistent with a European prevalence <0.07 per cent. Only Drosophila Vesanto virus and Drosophila Kallithea nudivirus seem relatively common—being detected in more than half of populations and having estimated prevalences of 2.9 and 2.1 per cent, respectively. It is unclear why DNA viruses should have such a low prevalence, on average, as compared with RNA viruses (Webster et al. 2015). In simple ‘susceptible-infected-susceptible’ compartment models, low pathogen prevalence can result from high lethality, low transmission rates, or high recovery rates (relative to baseline mortality rates). It is therefore possible that DNA virus infections are less persistent than RNA virus infections or that they have lower transmission rates. Alternatively, this may reflect sampling bias, such that DNA viruses increase morbidity to the extent that infected flies are less likely to be sampled than uninfected flies. Greater virulence may also explain why DNA viruses rarely persist through multiple generations in laboratory fly lines. Alternatively, it may be that the rare viruses represent dead-end spillover from other taxa that can only be seen here because of the large sample size. Third, although some viruses showed broad geographic patterns in prevalence, a lack of repeatability associated with sampling location, and the very high *F_ST_* values, hint that transient local epidemics may be the norm, with viruses frequently appearing and then disappearing from local fly populations.

Finally, *Drosophila* do indeed seem to harbour fewer DNA viruses than RNA viruses, supporting an observation that was made before any had been described (Brun and Plus 1980; Huszart and Imler 2008). This cannot simply be an artefact of reduced sampling effort, as almost all *Drosophila*-associated viruses have been reported from undirected metagenomic studies, and metagenomic studies of RNA are as capable of detecting expression from DNA viruses as they are of detecting RNA viruses (e.g. Webster et al. 2015). Instead, it suggests that the imbalance must reflect some aspect of host or virus biology. For example, it may be a consequence of differences in prevalence. If RNA viruses have higher prevalence in general, or specifically in those adult flies attracted to baits, and/or RNA viruses persist more easily in fly or cell cultures, then this may explain their more frequent detection.

Taken together, our analyses of the distribution and diversity of DNA viruses associated with *D.melanogaster* at the pan-European scale provide an ecological and evolutionary context for studies of host-virus interaction in *Drosophila*. However, we currently lack almost any data on the natural host range or fidelity of *Drosophila* viruses, and we have no knowledge of their real-world fitness consequences for the host. In the future, such information will be vital if we are to capitalise on *Drosophila* models to understand the co-evolutionary process.
